# Sarcopenia in daily practice: assessment and management

**DOI:** 10.1186/s12877-016-0349-4

**Published:** 2016-10-05

**Authors:** Charlotte Beaudart, Eugène McCloskey, Olivier Bruyère, Matteo Cesari, Yves Rolland, René Rizzoli, Islène Araujo de Carvalho, Jotheeswaran Amuthavalli Thiyagarajan, Ivan Bautmans, Marie-Claude Bertière, Maria Luisa Brandi, Nasser M. Al-Daghri, Nansa Burlet, Etienne Cavalier, Francesca Cerreta, Antonio Cherubini, Roger Fielding, Evelien Gielen, Francesco Landi, Jean Petermans, Jean-Yves Reginster, Marjolein Visser, John Kanis, Cyrus Cooper

**Affiliations:** 1 Department of Public Health, Epidemiology and Health Economics, University of Liège, Avenue Hippocrate 13, CHU B23, 4000 Liège, Belgium; 2 Centre for Metabolic Bone Diseases, University of Sheffield, Sheffield, UK; 3 MRC and Arthritis Research UK Centre for Integrated research in Musculoskeletal Ageing (CIMA), London, UK; 4 Gérontopôle, University Hospital of Toulouse, Toulouse, France; 5 INSERM UMR1027, University of Toulouse III Paul Sabatier, Toulouse, France; 6 Gérontopôle of Toulouse, University of Toulouse III, CHU Purpan, Toulouse, France; 7 Service of Bone Diseases, Faculty of Medicine, Geneva University Hospitals, Geneva, Switzerland; 8 World Health Organization, Geneva, Switzerland; 9 Gerontology and Frailty in Ageing Research Department, Vrije Universiteit Brussel (VUB), Brussels, Belgium; 10 Centre de Recherche et d’information Nutritionnelles, Paris, France; 11 Department of Surgery and Translational Medicine, University of Florence, viale Pieraccini 6, 59139 Florence, Italy; 12 Prince Mutaib Chair for Biomarkers of Osteoporosis, Biochemistry Department, College of Science, King Saud University, Riyadh, 11451 Saudi Arabia; 13 Department of Clinical Chemistry, University of Liège, CHU Sart-Tilman, Bât B35, 4000 Liège, Belgium; 14 Human Medicines Research and Development Support Division, Scientific Advice, London, UK; 15 Geriatrics and Geriatric Emergency Care, IRCCS-INRCA, Ancona, Italy; 16 Nutrition, Exercise Physiology and Sarcopenia Laboratory, Jean Mayer USDA Human Nutrition Research Center on Aging at Tufts University, Boston, USA; 17 Department of Geriatric Medicine, Katholieke Universiteit Leuven, Leuven, Belgium; 18 Department of Geriatrics, Neurosciences and Orthopedics, Catholic University of the Sacred Heart Rome, Milano, Italy; 19 Geriatric Department, CHU Sart-Tilman, Bât B35, 4000 Liège, Belgium; 20 Department of Health Sciences, VU University Amsterdam, Amsterdam, Netherlands; 21 Department of Nutrition and Dietetics, Internal Medicine, VU University Medical Center, Amsterdam, Netherlands; 22 Institute for Health and Aging, Catholic University of Australia, Melbourne, Australia; 23 MRC Lifecourse Epidemiology Unit, University of Southampton, Southampton, England UK; 24 NIHR Musculoskeletal Biomedical Research Unit, University of Oxford, Oxford, UK

**Keywords:** Sarcopenia, Daily practice, Assessment, Management, Tools

## Abstract

**Background:**

Sarcopenia is increasingly recognized as a correlate of ageing and is associated with increased likelihood of adverse outcomes including falls, fractures, frailty and mortality. Several tools have been recommended to assess muscle mass, muscle strength and physical performance in clinical trials. Whilst these tools have proven to be accurate and reliable in investigational settings, many are not easily applied to daily practice.

**Methods:**

This paper is based on literature reviews performed by members of the European Society for Clinical and Economic Aspects of Osteoporosis and Osteoarthritis (ESCEO) working group on frailty and sarcopenia. Face-to-face meetings were afterwards organized for the whole group to make amendments and discuss further recommendations.

**Results:**

This paper proposes some user-friendly and inexpensive methods that can be used to assess sarcopenia in real-life settings. Healthcare providers, particularly in primary care, should consider an assessment of sarcopenia in individuals at increased risk; suggested tools for assessing risk include the Red Flag Method, the SARC-F questionnaire, the SMI method or different prediction equations. Management of sarcopenia should primarily be patient centered and involve the combination of both resistance and endurance based activity programmes with or without dietary interventions. Development of a number of pharmacological interventions is also in progress.

**Conclusions:**

Assessment of sarcopenia in individuals with risk factors, symptoms and/or conditions exposing them to the risk of disability will become particularly important in the near future.

## Background

The term sarcopenia was first coined by Rosenberg et al. in 1989 [1] as a progressive loss of skeletal muscle mass with advancing age. Since then, the definition has expanded to incorporate the notion of impaired muscle strength and/or physical performance. Currently, several definitions of sarcopenia have been proposed [2–10] but no consensus has yet been reached. Depending on the definition used, the prevalence of sarcopenia is reported to be up to 29 % for older community-dwelling adults and up to 33 % for individuals living in long-term care institutions [11, 12]. Sarcopenia is associated with morbidity and mortality from linked physical disability, falls, fractures, poor quality of life, depression and hospitalization [13–19].

Current research is focusing on nutritional exercise/activity based and other novel interventions for improving the quality and quantity of skeletal muscle in older people. Some studies demonstrated that resistance training combined with nutritional supplements can improve muscle function [11, 20–22]. A number of pharmacological interventions are in development but no single agent has been shown to be clinically effective, without unwanted effects, in maintaining or increasing skeletal muscle mass or function. With the prospect of effective interventions, the identification and assessment of sarcopenia will become particularly important to prevent disability and other negative health outcome in the near future.

The challenge in clinical practice will be in the assessment of sarcopenia to identify those who might benefit most from these interventions. Among the l current definitions of sarcopenia [3, 7, 8], there is a general agreement on the need for muscle mass measurement with varying recommendations on the roles of muscle strength assessment and/or physical performance. Currently, several well validated tools exist to measure these parameters, which have been reviewed recently [18, 23, 24]. Whereas they have been used for sarcopenia case finding in the research setting, their use is not always feasible in daily clinical practice. The purpose of this paper is to discuss different approaches in the assessment of sarcopenia and potential management strategies in clinical practice.

## Methods

As in previous initiatives and publications [25–35], the European Society for Clinical and Economic Aspects of Osteoporosis and Osteoarthritis (ESCEO) working group on frailty and sarcopenia consists of clinical scientists and experts in the field of musculoskeletal diseases. Different members of the ESCEO working group were asked to prepare a review of the literature on 1) the general tools for the assessment of sarcopenia, both in research and in clinic (CC); 2) the assessment of physical performance in daily practice (MC); 3) the role of imaging in the diagnosis of sarcopenia in daily practice (MV); 4) the role of biochemical markers in the diagnosis of sarcopenia in daily practice (EC) and 5) the role of primary versus secondary care physicians in the evaluation of sarcopenia (AC). A brief summary of the management of sarcopenia in daily practice was also proposed and discussed. Randomized controlled studies, prospective studies, systematic reviews and meta-analyses published before September 2015 were searched on PubMed and Embase using the following search terms : 1) Sarcopenia, Clinical, Evaluation, Assessment, Management; 2) Physical function, Physical performance, Gait, Walk, Walking, Strength; 3) Elderly, Muscle mass, Sarcopenia, Dual x-ray absorptiometry/DXA/DEXA, Computer tomography/CT, Magnetic resonance imaging/MRI, Bioelectrical impedance/BIA; 4) Frailty, Sarcopenia, Biomarker, Biochemical marker, and 5) Primary care, Specialist care, Secondary care, Sarcopenia, Management, Screening, Questionnaire. Additional studies were identified by a manual search of bibliographic references of relevant articles and existing reviews. Each member prepared a list of the most important papers based on their review of the literature and then made a set of preliminary recommendations. The subsequent step was a face-to-face meeting for the whole group to make amendments and discuss further recommendations. The plan of the manuscript was also discussed and shared conclusions were reached. The views expressed in this article are the personal views of the authors and may not be understood or quoted as being made on behalf of or reflecting the position of the EMA or one of its committees or working parties.

## Results

### How to assess sarcopenia in clinical practice?

Despite a relatively large number of tools being available to measure muscle mass, muscle strength and physical performance [36, 37], some of them are likely to be of greater validity and utility for the assessment of sarcopenia in clinical practice than in clinical research and are summarised in Table 1. Whereas some biochemical markers of muscle metabolism (e.g. activin, n-terminal propeptide of procollagen III and myostatin) are being investigated for their ability to indicate muscle mass or strength, current data suggest that it is premature to recommend their use in daily practice [38].

**Table 1:** Applicability of the existing tools for the assessment of muscle mass, muscle strength and physical performance in research and clinical settings

	Applicable in research settings	Applicable in specialist clinical settings	Applicable in primary care settings
Assessment of muscle mass			
*DXA*	+++	+++	+
*Anthropometric measurements*	+	++	++
*CT-scan*	+++	++	+
*MRI*	+++	++	+
*BIA*	++	++	+
Assessment of muscle strength			
*Handgrip strength*	+++	+++	+++
*Lower limb muscle strength*	+++	++	+
*Repeated chair stands test*	+	+	++
Assessment of physical performance			
*Gait speed*	+++	+++	+++
*Timed Up and Go test*	++	+	+
*Balance test*	+	+	+
*6-min walk test*	++	+	+
*400 m walk test*	++	+	+
*Stair climb test*	++	+	+
*SPPB test*	+++	++	+

*SPPB* Short Physical Performance Battery

Nb. The group has chosen to attribute to each tool +++ (best recommended tool) or ++ (best alternative tool) or + (less recommended tool) based on the availability and the costs of the tool, the required time for the examination and the availability of robust cut-off points

There are currently a number of approaches to the definition of sarcopenia in clinical practice [3, 7, 36]. However, these are usually more suited to research studies than wider clinical practice. Additionally, some of the available methodologies for the assessment of sarcopenia utilise methods for measuring muscle mass, strength and physical function that are more suited to secondary care, than primary care settings. We therefore tabulated our preferences according to feasibility, complexity, required time for the examination, availability of robust cut-off points and cost, in each of these three contexts: research, specialist settings and primary care (Table 1).

#### Assessment of muscle mass

The widespread use of magnetic resonance imaging (MRI) and computed tomography (CT) scan for the non-invasive assessment of muscle mass [39] is limited in primary care settings by difficulties in access, costs, the lack of portable equipment and the requirement of highly specialized personnel.

Dual-energy x-ray absorptiometry (DXA) is a well-established, low-radiation technique used to assess body composition and provides reproducible estimates of appendicular skeletal lean mass [40, 41]. It is acknowledged that the accuracy of DXA for assessing muscle mass in people of different ages and different pathological conditions may vary. Moreover, DXA (in contrast to CT-scan and MRI) cannot assess intra-muscular fat, which turns out to be of increasing importance in terms of the quality of muscle and associations with clinical outcomes. Bearing these limitations in mind, DXA is still considered as the procedure of choice for routine clinical assessment. Using DXA, appendicular skeletal lean mass (ALM) is measured as the sum of the non-bone and non-fat mass of the four limbs. To adjust for body size, a skeletal muscle index (SMI) is derived as ALM/height^2^. Thresholds of SMI at two standard deviations below the mean SMI of young male and female reference groups have been proposed as gender-specific cut-off points for sarcopenia. This results in two thresholds, proposed by the EWGSOP [3], the first of 5.5 kg/m^2^ for women and 7.26 kg/m^2^ [8] for men and the second of 5.67 kg/m^2^ for women and 7.25 kg/m^2^ for men [42], depending on the reference group on which these cut-off have been established. Using a different approach, the FNIH sarcopenia project [7] has also recently defined cut-offs for appendicular lean mass adjusted for body mass index (BMI), giving values of < 0.512 for women and < 0.789 for men. However, it should be pointed that these cut-offs might also be modified according to ethnicity [43].

If clinicians have no access to DXA, they can use anthropometric measurements. Indeed, a recent survey [44] showed that anthropometric data are currently the most widely used methods in clinical practice (57.5 % of clinicians that measure muscle mass in their practice use anthropometric data) followed by DXA (45.9 %). Several anthropometric measurements exist (i.e. body mass index, calf circumference, mid-upper arm circumference and skinfold thickness). Moreover, mid-arm muscle and calf circumferences have been shown to be correlated with appendicular muscle mass and reflect both health and nutritional status and predict performance, health and survival in older people [45–47]. However, with advancing age, changes in the distribution of fat and loss of skin elasticity are such that circumference and skinfold measures incur a loss of accuracy and precision in older people [47, 48]. Some studies suggest that an adjustment of anthropometric measurements for age, sex or BMI results in a better correlation with DXA-measured lean mass [49–51]. Anthropometric measurements are simple clinical prediction tools that can be easily applied for sarcopenia since they offer the most portable, commonly applicable, inexpensive and non-invasive technique for assessing size, proportions and composition of the human body [50]. However, their validity is limited when applied to individuals due to large prediction errors and because cut-off points, to identify low muscle mass, still need to be defined. Therefore, if a patient is identified as at risk of having sarcopenia by anthropometric measurements, an additional measurement of muscle mass with DXA would still be recommended.

Finally, bio-electrical impedance analysis (BIA) is a method which estimates the volume of fat and lean body mass based on the relationship between the volume of a conductor and its electrical resistance. The method is not expensive, requires no specialized staff and is relatively easy to use in clinical practice, both on ambulatory subjects or on hospitalized patients. Moreover, reference values have been established for older individuals [3]. Even if the method’s accuracy has been challenged and has been reported to overestimate muscle mass and underestimate fat mass [52–54], it is possible to use some adjustment equations to obtain valid measurements [55].

*In summary, we would propose assessing primarily muscle mass by DXA, if this tool is available, and if not, anthropometry measurements can easily be used, in primary care settings, as a first screening of patients with low muscle mass. These patients can then be referred for an additional evaluation in specialist clinical settings.*

#### Assessment of muscle strength

Handgrip strength appears to be the most widely used method for the measurement of muscle strength. A recent survey indicated that clinicians, both from the fields of geriatric medicine and rheumatology, prefer the use of grip strength over chest press and lower limb isokinetic dynamometry as a measure of overall muscle strength [44]. In general, isometric handgrip strength shows a good correlation with leg strength [56] and also with lower extremity power, knee extension torque and calf cross-sectional muscle area [15, 57]. The measurement is easy to perform, inexpensive and does not require a specialist trained staff. Standardized conditions for the test [58] include seating the subject in a standard chair with their forearms resting flat on the armchairs. Clinicians should demonstrate the use of the dynamometer and show that gripping very tightly registers the best score. Six measures should be taken, 3 with each arm. Ideally, the patients should be encouraged to squeeze as hard and as tightly as possible during 3–5 seconds for each of the 6 trials; usually the highest reading of the 6 measurements is reported as the final result. The Jamar dynamometer, or similar hydraulic dynamometer, is the gold standard for this measurement. However, for patients with advanced arthritis, the design of this dynamometer may be a limitation [59]. A pneumatic dynamometer, such as the Martin vigorimeter, may be a good alternative. With this device, patients try to squeeze rubber balls (available in three sizes) with the same protocol as that used for the Jamar dynamometer. A variety of thresholds of grip strength have been proposed to characterize low muscle strength, ranging from 16 to 20 kg for women and 26–30 kg for men [7, 15, 60, 61]. Lower limb muscle strength, most frequently of the quadriceps, can also be measured. Commercial dynamometers can enable isometric and/or isokinetic measurements of strength. Even if these measurements are feasible in frail people [62, 63], they are often limited in clinical practice by their relative expense, the need to purchase dedicated equipment, the lack of trained staff and limited data in older populations. However, the repeated chair stand test, which is a timed test requiring participants to rise from a chair without using their arms and return to the seated position, consecutively, for five times, has been shown to be able to provide a reasonably reliable and valid indication of lower body strength [64].

*In summary, we would recommend to measure muscle strength by handgrip strength in clinical practice* (Table 1)*. For primary care settings where the availability of a handgrip dynamometer is not systematic, the repeated chair stand test could be used as an alternative measure of muscle strength.*

#### Assessment of physical performance

The most widely used tool in clinical practice for the assessment of physical performance is the gait speed measurement, employed by almost two-thirds (63.3 %) of clinicians that assess physical performance (among 255 clinicians who took part in an international online survey; 87.8 % of medical doctors with geriatrics (57.6 %) and rheumatology (18.8 %) as major fields of interest) [44]. The test is highly acceptable for participants and health professionals in clinical settings [65]. No special equipment is required as it only needs a flat floor devoid of obstacles. In the 4-m gait speed test, which is recommended by the EWGSOP for the assessment of sarcopenia, men and women with a gait speed <0.8 m/s are described as having a poor physical performance [15]. The average extra-time added to the consultation by measuring the 4-metre gait speed was only 95 ± 20 s.

Gait speed can be performed alone or as part of a test battery, the most popular of which is the Short Physical Performance Battery (SPPB). The SPPB is a test scored to a maximum of 12 points comprising an assessment of gait speed (over 3–4 m), a balance test and a repeated chair stand test. These tests focus on lower extremity function, as the latter has been shown to correlate with mobility, disability and patient outcomes including hospitalization, institutionalization, and mortality. The SPPB takes about 10 min to complete [66]. Participants presenting a score ≤8 points have been described as having a poor physical performance [3].

Other standalone tests can be performed to assess physical performance. In the Timed Up and Go (TUG) test, individuals are asked to rise from a standard armchair, walk to a marker 3 m away, turn, walk back and sit down again. The 6-min walk distance or 400 m walk time can be used to measure aerobic capacity. The stair climb power test also shows good correlation with other measures of leg power and physical performance, but is mostly restricted to use in research settings [67].

*In summary, we would propose that physical performance is primarily assessed in clinical practice by measuring gait speed. The SPPB test may be limited by the time of administration but might also be useful to identify men and women with low physical performance* (Table 1).

### The role of primary care physicians

In view of the current lack of a consensus concerning the definition of sarcopenia and also of the practical issues related to time constraints and limited access to assessment tools in the primary care setting, the group believes that the role of primary care physicians should be to identify patients who are at risk of sarcopenia and to refer them to specialists in the field. Some interesting methods that might be suitable for screening purpose are presented in the following section.

Consideration of possible sarcopenia should be undertaken in older individuals (e.g. > 65 years) with signs or symptoms suggestive of the condition both in primary care and in specialized clinical settings. Several methods can be proposed to perform a simple, rapid and inexpensive identification of those at risk. However, none of them has received an extensive validation, and therefore further research in this area is urgently needed.

#### The red flag method

The purpose of the red flag method is to understand, during a standard medical consultation (or health assessment) the clinical presentation of individuals with particular regard to physical manifestations of sarcopenia such as general weakness or loss of muscle mass. The subject can also be asked about symptoms such as loss of weight, loss of muscle strength, loss of energy, falls, etc. (Table 2). An assessment of nutrition habits should also be performed to check, for example, if the subject has sufficient protein intake. The Mini-Nutritional Assessment could also be used for a rapid and easy assessment of malnutrition or, at least, risk of malnutrition [68]. Finally, clinicians can also assess physical activity. Indeed physical inactivity or high levels of sedentary behaviour may be considered a red flag. If the screening identifies any red flag suggesting the presence of sarcopenia, more sophisticated assessment procedures of sarcopenia can be implemented. Red flags have been identified through reviewed papers identified by members of the group and are presented in Table 2.

**Table 2:** The Red Flag method

	Red flags
Clinician’s observation	General weakness of the subject
	Visual identification of loss of muscle mass
	Low walking speed
Subject’s presenting features	Loss of weight
	Loss of muscle strength, in arms or in legs
	General weakness
	Fatigue
	Falls
	Mobility impairment
	Loss of energy
	Difficulties in physical activities or activities of daily living
Clinician’s assessment	Nutrition
	Body weight
	Physical activity

Nb. Red flags have been identified through reviewed papers identified by members of the group

#### The SARC-F questionnaire

The SARC-F questionnaire [69] was developed as a possible rapid screening test for sarcopenia. This questionnaire could enable healthcare providers to quickly and easily assess the risk of sarcopenia during a standard health consultation. The subject is asked 5 questions addressing strength, assistance in walking, rising from a chair, stair climbing and falls. Each component is scored from 0 to 2 points, giving a global score of the SARC-F between 0 and 10 points. A score ≥ 4 points is reported to be predictive of sarcopenia and poor outcomes and could be a trigger for a more detailed assessment of sarcopenia.

Despite a questionable sensitivity [70], the SARC-F questionnaire is considered as one of the best available tools to be used in primary care for raising awareness of the diagnosis of sarcopenia. Similarly to the red flag method, a result ≥ 4 for the SARC-F questionnaire could be an incentive to send the subject to a complete assessment of sarcopenia.

#### Prediction of low muscle mass according to age and BMI

Recently, a study [71] has been performed with the purpose to identify predictors of low skeletal muscle mass in older adults toward development of a practical clinical assessment tool for use by clinicians to identify individuals requiring DXA screening for muscle mass. For this purpose, ALM was calculated from DXA scans and SMI defined as the ratio of ALM divided by height in square centimetres. Older participants (from 65 to 85 years) were classified has having low muscle mass if their SMI was 1 standard deviation below the mean SMI of young adults. This model was validated on a sample of 200 subjects of the NHANES population. Results of the validation analysis revealed that age and BMI were strongly associated with a low SMI and may be an informative predictor in the primary care settings. Consequently, two models were proposed, one for men and one for women and consist of two tables presenting the probability of low muscle mass by age and BMI. In a 200-person validation, the model sensitivity was 81.6 % for men and 90.6 % for women and the model specificity was 66.1 % for men and 66.2 % for women.

#### Anthropometric prediction equation in combination with a measure of muscle function

Other authors developed gender specific anthropometric equations, based on age, weight, BMI values, to estimate appendicular skeletal muscle mass [72]. To validate these prediction equations, muscle mass was assessed using DXA in three cohorts of older Australian subjects [72] (appendicular skeletal muscle mass prediction equation: 10.05 + 0.35(weight) − 0.62(BMI) − 0.02(age) + 5.10 (if male)). The results showed a strong correlation between the equations and the muscle mass measured using DXA, with an adjusted R^2^ of 0.869. In a subsequent research the prediction equations were evaluated in combination with assessment of hand grip strength as a screening method to identify older patients who should undergo DXA evaluation for sarcopenia. The best strategy to reduce the number of DXA was to apply the equation first, to assess hand grip strength in those with low estimated muscle mass and to proceed to DXA only in individuals with low grip strength [50].

#### Prediction of sarcopenia using age, handgrip strength and calf circumference

In 2014, Ishii et al. [73] developed a new screening tool for sarcopenia in a sample of almost 2000 autonomous community-dwelling older subjects in Japan. Sarcopenia was defined on the basis of low muscle mass measured by BIA and either low muscle strength characterized by handgrip or low physical performance characterized by slow gait speed. Using a database including demographic variables, albumin, chronic diseases, physical activity information and anthropometric measurements, the authors developed a gender specific model including three variables, i.e. age, handgrip strength and calf circumference. Based on the model, the authors constructed a gender specific score chart that had an excellent discrimination ability, with an area under the curve of 0.939 for men and 0.909 for women. The formula to calculate the scores are as follows: score in men, 0.62 x (age-64) – 3.09 x (grip strength −50) – 4.64 x (calf circumference −42); score in women, 0.80 x (age-64) – 5.09 (grip strength −34) – 3.28 x (calf circumference – 42). The corresponding probabilities of sarcopenia were calculated as: probability in men, 1 / [1 + e^-(sum score/10–11.9)^]; probability in women, 1/[1 + e ^-(sum score/10–12.5)^]. This model still requires further validation in independent cohorts, before its use in clinical practice can be promoted.

### How to manage sarcopenia in daily practice?

#### Identification of comorbidities

Sarcopenia is frequently found in association with comorbidities, e.g. osteoporosis, osteopenia, obesity, type II diabetes mellitus, breast cancer, etc. [74, 75]. In such cases, sarcopenia may be considered as a secondary consequence of the co-existing pathological condition. The impact of management of these conditions (e.g. better diabetic control, reduction of inflammatory status, or weight loss in obesity due to an energy-restricted diet) on the accompanying sarcopenia is unclear [76].

#### Physical activity

Physical activity interventions and progressive resistance training have been suggested to have a predominant effect on muscle strength, muscle mass and physical performance in older people [77].

However, so far, studies mainly focusing on well-defined sarcopenia with standardization of the physical intervention are still missing. Hence, it is still difficult to give a patient-specific physical activity prescription for the management of sarcopenia. However, healthcare providers can nevertheless give some general recommendations in order to improve other common conditions in older adults (WHO recommendation: http://www.who.int/dietphysicalactivity/factsheet_olderadults/en/). Moreover, in their review, Cruz Jentoft at al [11]. forwarded two recommendations regarding the management of physical activity interventions in older people. First, to obtain an impact on muscle function, the duration of the intervention should be for at least 3 months. Second, supervised resistance exercise or multicomponent/combined exercise programs should be recommended for frail or sedentary community-dwelling people.

#### Nutrition

Although nutrition is considered as a major point for the management of sarcopenia, evidence of the effect of nutrition on muscle function is often derived from short-term studies in specifically selected sample and large clinical trials are still lacking. Currently, there is no robust evidence for nutritional recommendations for subjects with sarcopenia.

However, even if randomized controlled trials are inconsistent regarding the effects of protein supplementation on muscle function, several observational studies have suggested that maintaining adequate protein intake may help preserve muscle mass and strength in both adults and older people [78, 79]. Bauer et al. [80] recommended increasing protein intake to 1.2 g/kg body weight/day either by diet or by protein supplementation in older adults because of blunted muscle protein synthetic response and blunted post-prandial inhibition of muscle protein breakdown (anabolic resistance). Frail older adults or older who have acute or chronic diseases need higher dietary protein (i.e. 1.2–1.5 g/kg body weight/d) [80]. Recent evidence suggests that the recommended dietary allowance for protein is inadequate in older people [81]. Some other nutritional supplements, such as β-hydroxy β-methylbutyrate, creatine and vitamin D have been suggested to have an effect on muscle function. Indeed, β-hydroxy β-methylbutyrate supplements appear to increase muscle mass whilst its effects on muscle strength and physical performance are inconsistent [11, 20, 21]. Supplementation with creatine, protein or leucine combined with resistance exercises seems to have a positive impact on muscle mass, muscle strength and physical performance [22, 82, 83]. Finally, a recent meta-analysis has suggested that vitamin D supplementation could increase lower limb muscle strength [84]. Based on this evidence, dietary protein caloric intake, protein quality, as well as the vitamin D status of older individuals could be checked by clinicians and/or dieticians and individual prescription of nutritional supplements could be considered.

#### Pharmacological management

Currently, no drug is registered for the treatment of sarcopenia. However, several new chemical entities are currently at various stages of development. These are summarized in Table 3 with their potential future indications and their current phase of development.

**Table 3:** Pharmacological agents in development with potential for treating sarcopenia

Mechanism of action	Drug name	Drug Developer	Indication sought	Study phase
I. Myostatin Antagonists				
Activin receptor trap	ACE-031	Acceleron	Duchenne muscular dystrophy	Phase 3 (trial terminated early)
Myostatin antibody	REGN-1033	Regeneron/Sanofi	Sarcopenia	Phase 2
	LY-2495655	Eli Lilly	Hip arthroplasty	Phase 2
			Elderly Fallers	
			Cancer Cachexia	
	PF-06252616	Pfizer	Inclusion body myositis	Phase 1
Activin receptor inhibitor	Bimagrumab (BMY338)	Novartis	Sarcopenia	Phase 2 and 3
			Hip fracture	Phase 2
			Cancer and COPD cachexia	
II. Selective Androgen Receptor Modulators	Enobasarm (Ostarine)	GTx	Cancer Cachexia	Phase 3 (did not meet primary endpoint)
III. Skeletal Troponin Activators	Tirasemtiv	Cytokinetics	ALS	Phase 2,3
	CK-2017357		Myasthenia Gravis	

## Discussion and general consensus

The ESCEO Experts group agreed on some general recommendations to be implemented in clinical practice:

- Several tools are currently available for the measurement of muscle mass, muscle strength and physical performance, with a potential use for the diagnosis and follow-up of sarcopenia but they are not fully adapted for widespread use in clinical daily practice. The recommended tools for the diagnosis of sarcopenia in specialist clinical practice are DXA for the measurement of appendicular muscle mass, grip strength for the measurement of muscle strength and gait speed for the measurement of physical performance. Thresholds previously recommended in the literature can be applied to distinguish normal from abnormal;
- Healthcare providers, particularly in primary care, should consider an assessment of sarcopenia in individuals at increased risk; suggested tools for assessing risk include the SARC-F questionnaire, the SMI method or different prediction equations based on anthropometric data associated with the measurement of handgrip strength, although all of them require further validation;
- Whereas further studies are required to provide a full evidence-based guidance to clinicians, current management can include physical activity advice, particularly progressive resistance training, treatment and prevention of vitamin D deficiency and adequate energy and dietary protein intake.

The Expert group also emphasizes the importance of education and increased awareness of clinicians to the potential deleterious outcomes of sarcopenia.

## Conclusions

Physicians and other health professionals have an important role to play in the assessment and management of sarcopenia to reduce its impact on individuals’ well-being, the development of disability, and on health resources utilization.
